# Channel based generating function approach to the stochastic Hodgkin-Huxley neuronal system

**DOI:** 10.1038/srep22662

**Published:** 2016-03-04

**Authors:** Anqi Ling, Yandong Huang, Jianwei Shuai, Yueheng Lan

**Affiliations:** 1 Department of Physics, Tsinghua University, Beijing 100084, China; 2 Collaborative Innovation Center of Quantum Matter, Beijing 100084, China; 3 Department of Physics and Institute of Theoretical Physics and Astrophysics, Xiamen University, Xiamen 361005, China

## Abstract

Internal and external fluctuations, such as channel noise and synaptic noise, contribute to the generation of spontaneous action potentials in neurons. Many different Langevin approaches have been proposed to speed up the computation but with waning accuracy especially at small channel numbers. We apply a generating function approach to the master equation for the ion channel dynamics and further propose two accelerating algorithms, with an accuracy close to the Gillespie algorithm but with much higher efficiency, opening the door for expedited simulation of noisy action potential propagating along axons or other types of noisy signal transduction.

Hodgkin and Huxley first proposed a classical way to deterministically characterize neuronal dynamics based on a quantitative analysis of experimental results^1^. The phenomenological Hodgkin-Huxley equations treat the neuron membrane as a capacitor with a set of continuous parallel channels for the passage of ions, and the permeability of the neuronal membrane determines ion-specific currents. Each ion channel has four subunits, being independent and each either open or closed. The conductance is determined by the fraction of ion channels in an open state in which all subunits are open. With further indepth investigation, stochasticity is found to play an important role in neuronal dynamics^2^,^3^,^4^ and the cooperative behaviors in biological neuronal networks, such as pattern formation^5^, synchronization^6^,^7^,^8^ and coherence^9^,^10^,^11^ which are of great importance to the understanding of generation and functioning of several neural diseases^5^. Individual voltage- or ligand-gated ion channel randomly alternates between open and closed states which turns out to be a major source of noise in neuronal activity, the so-called channel noise^12^,^13^, and contributes to the generation of spontaneous action potentials^14^,^15^, variability in spike timing^16^, firing coherence^10^, and the regularity of spontaneous spiking activity^11^. The nonlinear amplification of synaptic signal makes up another source of noise, which also greatly influences the membrane potential fluctuations^17^,^18^,^19^,^20^.

Channel noise has been identified to be essential to neuronal dynamics and coding, and has been extensively studied in recent years in a variety of neural systems, like the auditory nerve by cochlear implants^21^, and in cerebellar granule cells^22^. It turns out that channel noise has measurable effects under normal conditions, which not only makes a big difference to the initiation and propagation of action potentials, such as firing irregularity^23^,^24^, spiking threshold and firing rate^25^,^26^, but also enhances sub-threshold signal above certain magnitude^27^.

As mentioned above, ion channels are subject to random changes among a number of possible channel conformations. The stochastic kinetics of ion channels could be defined as a Markov chain, with discrete phase space states where each state in the chain represents a particular configuration of the ion channel. The random transition of an ion channel from one state to another just depends on its current state in the Markov assumption, which can be exactly simulated by the Gillespie algorithm^28^. This algorithm tracks the number of channels in each state at each time point on one trajectory, and many trajectories are computed for well converged statistics. It is both accurate and simple to use but computationally demanding especially in the large channel number limit and hard to analyze mathematically which may be essential for an indepth understanding of neuronal dynamics.

One commonly used alternative is the Langevin approach which treats channel noise as a Gaussian one, first proposed by Fox and Lu^29^,^30^. However, in comparison with the exact Gillespie algorithm, the original Langevin approaches could not accurately capture the stochastic channel dynamics. One computes subunit fractions with noise in which the subunits of *K*^+^ and *Na*^+^ channels are identical (Identical LA)^29^,^30^. An improvement was put forward through rescaling the noise intensity with an empirical factor by Huang *et al*. (Rescaled LA)^31^. Another one adds noise to the channel fractions (Fox-Lu channel based LA)^29^. Nevertheless, the channel fractions obtained from the corresponding numerical computation may be out of the biologically meaningful interval [0, 1], especially at small channel numbers. The Cholesky decomposition was used to treat the stochastic terms (Orio LA)^32^, known as channel-based Langevin approach with unbounded state fractions. To bound the channel state fractions within [0, 1], a reflection boundary condition was supplied^33^. A more accurate version was later proposed by Huang *et al*., who designed a restoration scheme to put the changes of state fractions back to the SDEs after a truncation (Truncated-Restored LA)^34^. It has been pointed out that the bounded Truncated-Restored Langevin approach and the unbounded Orio Langevin approach exhibit equally good, and also the best approximations to the exact Markov dynamics among these Langevin approaches^35^. However, as reviewed by Huang *et al*., despite for small channel numbers, currently proposed Langevin approaches cannot accurately replicate the statistical properties of the Markov HH model even at large channel numbers, calling for a better approach to the stochastic HH dynamics^35^.

Nevertheless, a Markov chain is completely described by a master equation, a group of ordinary differential equations for the probabilities of discrete states^36^. There are many approximate approaches developed for solving master equations in a variety of biochemical networks, for instance, the noisy signal transduction network^37^,^38^,^39^. Lan *et al*. demonstrated the equivalence of the field theoretic formulation to the generating function approach which is based on mapping an enormous set of master equations (ODEs) into one single partial differential equation (PDE)^40^, and has been applied in a plethora of cases^41^. In the current work, we design a new hybrid scheme for the computation of noisy neuron dynamics based on the generating function formulation, and in addition propose two accelerating algorithms for improved efficiency. Our method produces statistics of stochastic action potential that agree well with exact ones in different situations no matter if the channel number is small or large, the input current is constant or noisy. It is computationally efficient compared to the Gillespie algorithm and many variants of Langevin approach. When the channel number is small, it produces results that match those from Gillespie computation while all Langevin approaches fail or are not accurate. The current approach balances well accuracy and efficiency and opens the door for large-scale computation in stochastic axonal dynamics.

## Results

### Implementation with generating function

As discussed in the Methods section, the propagation of the action potential along an axon is described by a stochastic version of the HH equation, which could be transformed to a PDE for the generating function of the channel system. As the transition rates of the *K*^+^ channel state depends on *V*, the expression below is an analytic approximate solution of the generating function equation


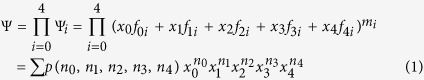


which would become exact if the time dependence of *V* has been known. (*n*_0_, *n*_1_, *n*_2_, *n*_3_, *n*_4_) denotes one possible distribution of the *K*^+^ channels with the probability *P*(*n*_0_, *n*_1_, *n*_2_, *n*_3_, *n*_4_). Here *m*_*i*_ is the number of channels in the *i*th channel state at *t* = *t*_0_ and the probability *f*_*ij*_ satisfies the equation


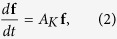


where *A*_*K*_ is the transition matrix of the *K*^+^ channel states.

A similar expression can be written down for the *Na*^+^ channel


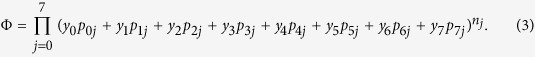


Here *n*_*j*_ is the number of *Na*^+^ channels in the *j*th state at *t* = *t*_0_ and *p*_*ij*_ satisfies the equation


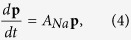


where *A*_*Na*_ is the transition matrix of the *Na*^+^ channel states, and *p*_7*j*_ is the probability that during the evolution time the *j*th *Na*^+^ channel state makes a transition to the 7^th^ channel state, i.e., the open state.

The mean value and standard deviation of the number of open *K*^+^ and *Na*^+^ channels are computed from the generating function:


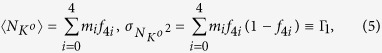



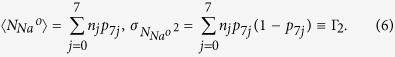


Since 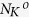, 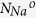 are random variables, the voltage *V* is also random. But, we could speculate that the distribution of *V* would be narrow during a small time interval if starting with a definite value. However, with time evolution the distribution of the random variable *V* becomes wider and wider. If *σ*_*V*_ which is the standard deviation of V is larger than a given width, then we should sample a new set of numbers {*m*_*K*_}, {*m*_*Na*_} to describe the *K*^+^ and *Na*^+^ channel states according to the probability distribution given by the generating function, and reinitialize *f* and *p*. In this way, *V* could be always approximated by the local 〈*V*〉 at any time.

In the Methods section, a linear noise approximation is used to estimate *σ*_*V*_. A new sampling is made whenever *σ*_*V*_ > (*D*_*V*_)_*T*_, where (*D*_*V*_)_*T*_ is a chosen membrane voltage threshold. Specifically, sampling of *K*^+^ and *Na*^+^ states according to the multi-nomial distribution, based on the probabilities given by *f* and *p*, may be decomposed into successive binomial ones. Take the sampling of *K*^+^ states for example. First, we could sample the number *m*_*i*4_ of which the *i*th channel state makes a transition to the 4th, i.e., the open state, with the transition probability *f*_4*i*_. After this has been done, Ψ_*i*_ becomes 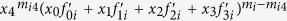, where 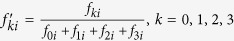.

Similarly, one by one we could get the transition numbers from the *i*th to the 0, 1, 2, 3th channel state respectively. After all samplings are done, Ψ_*i*_ will be 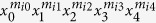. The generating function of the sampled state would be 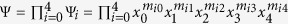, and we could get a distribution of the *K*^+^ channel state 
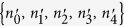
, where 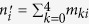. The sampling of *Na*^+^ states could be done in a similar way.

Besides that, the sampling of membrane voltage may be needed. The assumption that *V* behaves according to a random normal distribution with mean 〈*V*〉 and standard deviation *σ*_*V*_ seems reasonable. Nevertheless, according to our experience, it is comparatively convenient to fix the width of the distribution as taking *dV* = 0.1 *mV* in the sampling of *V*.

As a summary, we arrive at the following procedure:

1. Initialize the number of each *K*^+^ and *Na*^+^ channel state {*m*_*K*_}, {*m*_*Na*_} at time *t*, and set *f* and *p* the identity matrix.
2. Solve the ODEs for *f*, *p*, 〈*V*〉, and *σ*_*V*_.
3. Integrate to time *t*′ until the inequality *σ*_*V*_ ≤ (*D*_*V*_)_*T*_ breaks, sample a new set of channel numbers in each *K*^+^ and *Na*^+^ channel state with the probabilities computed from the generating function, and sample a new *V* based on the normal distribution *N*(〈*V*〉, *σV*). Take *σ*_*V*_ to be 0 and reinitialize *f* and *p*.
4. Check if *t* ≥ *T*. Yes, stop computation. No, go back to (2).

Though the above scheme is precise and can fully capture the dynamics of the system, it involves many ODEs and during the evolution many channel numbers will be sampled, so that the calculation is cumbersome. We propose two approximations to accelerate the algorithm under the premise of accuracy. Accelerating algorithm 1 is designed to reduce samplings of channel numbers. In accelerating algorithm 2, even the number of ODEs is much reduced.

### Accelerating algorithm 1

As *V* just depends on the number of open ion channels, we need only to sample the open channel numbers with the known transition probability of each state to the open state when the voltage width limit is reached at step (3) above. Take the *K*^+^ channel as an example. After we sample open channel numbers as discussed above, the generating function turns to


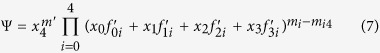


where *m*′ = *m*_04_ + *m*_14_ + *m*_24_ + *m*_34_ + *m*_44_ is the total number of open potassium channels.

As the probability flow in 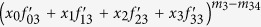 is close to that in 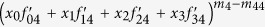 and *m*_4_ − *m*_44_ is small, one can take the critical step to merge the two factors 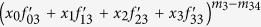

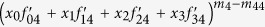
 into one 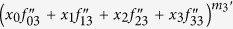, where 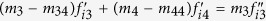, and 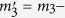 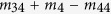, which determines 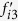 in a unique way.

### Accelerating algorithm 2

If the channel number is large enough, a reasonable approximation to binomial distribution is given by a normal distribution. And as we know, if X and Y are independent random variables that are normally distributed, then their sum is also normally distributed, with its mean being the sum of the two means, and its variance being the sum of the two variances. So, if all the channel numbers are large, we can take the generating function as (*x*_0  _*f*_0_ + *x*_1  _*f*_1_ + *x*_2  _*f*_2_ + *x*_3  _*f*_3_ + *x*_4  _*f*_4_)^*m*^, where m is the total number of *K*^+^ channel. In this case, as above, we can just sample the number in the open channel state when the voltage width limit is reached at above step (3), and the generating function turns out to be 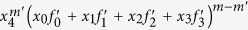 after the sampling. As time goes by, after the next sampling the generating function becomes 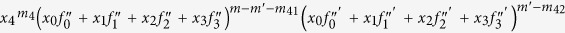, where *m*_4_ = *m*_41_ + *m*_42_. Same as accelerating algorithm 1, the latter two terms have similar probability flow, and can be merged into one multi-nomial form.

Similar procedure could be applied to the *Na*^+^ channel.

Accelerating algorithms 1 and 2 need only to sample the open ion channels, while the full generating function approach updates all. What’s more important is that the number of variables of the ODEs for *f* and *p* is much reduced in algorithm 2 which further reduces the computation time compared to algorithm 1. Hence, we would use the accelerating algorithm 2 to effectively reduce computation load.

Here we compare statistics of observables obtained from the generating function approaches with those obtained from the Gillespie algorithm and five Langevin approaches (Identical LA, Rescaled LA, Fox-Lu channel based LA, Orio LA, Truncated-Restored LA). The number of *Na*^+^ channels maintains three times that of *K*^+^ channels in the simulation. Data are saved at every 0.01 ms.

### Open Channel statistics

When the membrane voltage is fixed at a preset level, we measure the electric current and check how the neuron reacts to changes with different membrane potentials^42^. Here we compute the means and standard deviations of the open fraction of sodium and potassium channels under voltage clamp to study the dependence on membrane-patch voltage *V*. They were calculated over 100000 realizations using the Gillespie algorithm and Langevin approaches, each simulation was run for a total of 100 ms. The numbers of open sodium and potassium channels are collected at the final time point.

**Figure 1:**
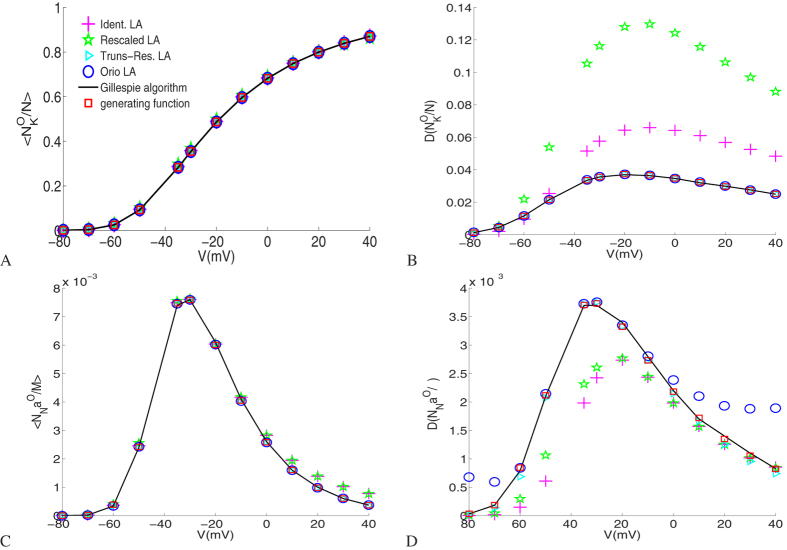
The means and standard deviations of the fraction of open channels with fixed membrane voltage. Results for the *K*^+^ channel (**A**,**B**) and for the *Na*^+^ channel (**C**,**D**). The total number *N* of the *K*^+^ channel is 180, and of the *Na*^+^ channel *M* = 540. The current *I* is set to 0 *uA*/*cm*^2^.

Figure 1 shows that as membrane voltage increases, the mean open fraction of the *K*^+^ channel increases and saturates at 1, while that of the *Na*^+^ channel increases first and then decreases, maximizing at *V* ~ −30 *mV*. As mentioned above, the opening and closing rates for “activation” subunits and “inactivation” subunits respond oppositely to the change in membrane voltage. As membrane voltage increases, the transition to the open state becomes fast with four identical *n* subunits in any *K*^+^ channel. But the activation of three *m* subunits and one *h* subunit works heterogeneously to jointly determine the channel state of *Na*^+^ channel, so that the transition rate does not monotonically increase.

We see from Fig. 1 that the mean fractions of open channels calculated from the four Langevin approaches are quite similar to the results obtained from the exact Gillspie algorithm. The Truncated-Restored LA replicates the standard deviation, whereas, the standard deviation of potassium currents is overestimated by the two subunit-based LAs, and the standard deviation of sodium currents is underestimated by the two subunit-based LAs and the Truncated-Restored LA, while overestimated by Orio LA. Obvious deviations from the exact ones are found in the two subunit-based LAs.

The results obtained by the generating function approach and by the Gillespie calculation match very well with each other not only for the mean fractions of open channels but also for their deviations. Indeed, under voltage clamp, the generating function result is exact. All subunits in the *K*^+^ channel are identical by assumption, we can show that the generating function will be a multi-nomial (*x*_0  _*f*_0_ + *x*_1  _*f*_1_ + *x*_2  _*f*_2_ + *x*_3  _*f*_3_ + *x*_4  _*f*_4_)^*N*^, if starting from a particular state, where *N* is the total number of *K*^+^ channels. Hence, the mean value and the standard deviation of open *K*^+^ channels are given by eq. (5). The stationary probability distribution is derived from


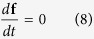


which gives the mean and standard deviation of the open *K*^+^ channel as 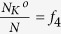 and 
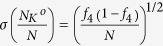
, where *f*_4_ is 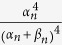. Similarly, the stationary probability distribution for the *Na*^+^ channel is calculated from


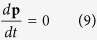


which gives the mean and standard deviation of the open *Na*^+^ channel as 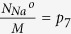 and 
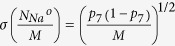
, where 
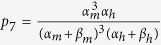
.

It can be concluded that the mean fractions of open channels are independent of the total channel numbers, and the standard deviations are proportional to 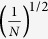 or 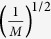, while these quantities depend on the voltage *V* implicitly.

### Spike statistics with no current input

Neuronal signaling study involves measuring and characterizing how stimulus signal propagates, indicated by the neuron action potentials or spikes. Particularly, the statistical study of action potentials is vital to describe and analyze neuronal firing. For example the lengths of interspike intervals (ISIs) often vary randomly but encode important information^43^.

An action potential is triggered once the voltage is beyond a threshold *V*_*T*_ for the deterministic HH neuron, giving a spike. Here *V*_*T*_ = −60 *mV* is considered. The spike amplitude *H* is defined as the difference from the peak voltage to the threshold in membrane potential. Considering the absorption of action potentials in the stochastic channel dynamics, a fully developed spike is identified only when the voltage goes up to at least −30 *mV* after going beyond the threshold *V*_*T*_, and shoots back downward below the resting level. That is, the spike has a minimal amplitude *H*_0_ to be 30 *mV*. The interspike interval *T* is defined as the time interval between two successive spike peaks.

**Figure 2:**
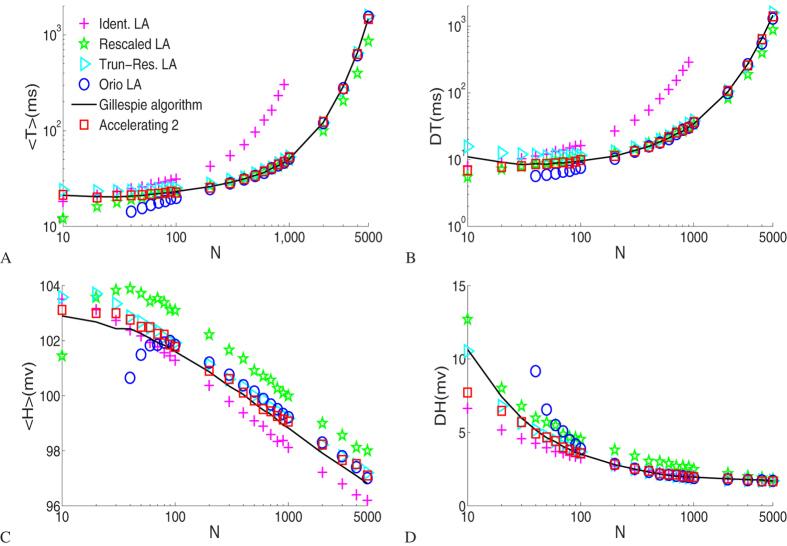
Comparison of the means and the standard deviations of first 10000 interspike intervals and spike amplitudes as a function of the total number of potassium channels, where *I* = 0 *uA*/*cm*^2^.

Figure 2 shows the statistical properties of the first 10000 successive spikes for *N* ranging from 10–5000, in which the input current *I* is 0. These simulations show that the rates and amplitudes of spiking activity obtained in the accelerating algorithm 2 and the Gillespie algorithm match well. Spiking events become increasingly rare as channel number increases, because the membrane dynamics tends to converge to its deterministic limit gradually in which large voltage fluctuations become increasingly rare while spiking events are mainly driven by large channel noise, and spike amplitude tends to decrease.

We can see from Fig. 2A that the spiking rates obtained from the accelerating algorithm 2 and from the Gillespie algorithm match well at both small and large channel numbers. The threshold for the voltage fluctuation controls the spike frequency and should be carefully chosen in the generating function scheme. The mean ISIs from the Rescaled LA tend to be much shorter than those from the Gillespie algorithm, while in the Identical LA they tend to be longer when channel number *N* > 40 and much longer at big channel numbers. A possible source for these differences between the two subunit-based Langevin approaches is that the noise term added to the subunit fractions should not be of the uncorrelated, zero mean Gaussian type. The results produced by the two channel-based Langevin approaches are in good quantitative agreement with those by the Gillespie algorithm. Nevertheless, there are still some discrepancies at small channel numbers, and the Orio LA breaks down at channel number less than 40. In general, other than the Identical subunit LA, the mean amplitudes calculated from these methods are bigger than that from the Gillespie algorithm, while the discrepancies are minimal between the results from accelerating algorithm 2 and the Gillespie algorithm.

### Interspike interval statistics with noisy current input

We study the spiking response to synaptic noise and discuss the statistics of the interspike intervals under a range of current conditions. The noisy current *I* is of the simple form *I*_0_ + *I*_1_*ξ* 44 here, where *ξ* is a Guassian white noise with zero mean and unit variance. *I*_0_ refers to the DC level. Due to the additive white noise term *I*_1_*ξ* which represents the combined effect of a continuous barrage synaptic noisy inputs that neurons in cortical and other neural systems receive, the action potentials may become more fluctuating^44^.

**Figure 3:**
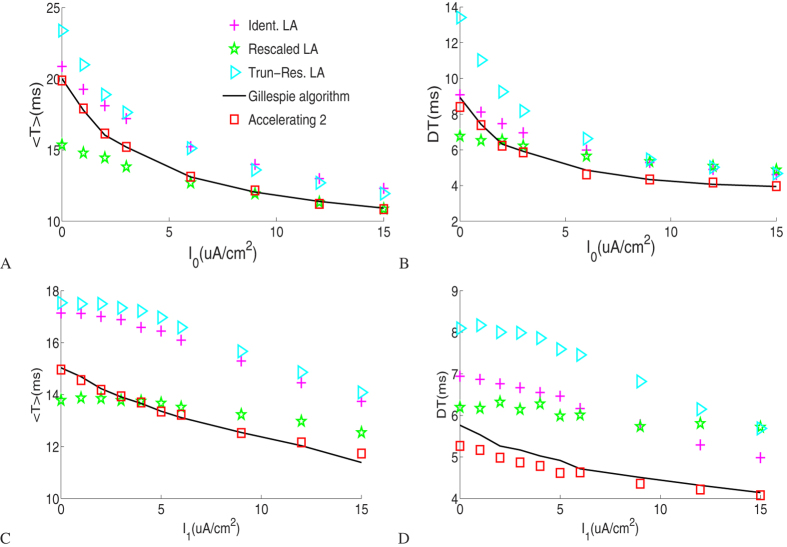
(**A**,**B**) Comparison of the means and standard deviations of the first 10000 interspike intervals as a function of *I*_0_ for *I*_1_ = 0 *uA*/*cm*^2^. (**C**,**D**) Comparison of the means and standard deviations of the first 10000 interspike intervals as a function of *I*_1_ for *I*_0_ = 3 *uA*/*cm*^2^. Here the total number *N* of *K*^+^ is set to 18.

Figure 3 plots the means and standard deviations (left and right columns, respectively) of ISIs with different noisy current levels for the Langevin approaches, Gillespie algorithm and accelerating algorithm 2 with small potassium channel number *N* = 18. As a fact, at such a small channel number, the Orio LA breaks down. As the DC level is increased, spiking events become more frequent as shown in Fig. 3A where there is no synaptic noise input and the channel noise reduces its impact on spike timing. Likewise, under a constant DC input, spiking rate increases with the increase of the intensity of the synaptic noise as Fig. 3C suggests. We see that the means and the standard deviations of ISI given by the Gillespie algorithm and accelerating algorithm 2 for different current inputs agree extremely well. Nevertheless there are still some discrepancies between the Langevin approaches and the Gillespie algorithm.

**Figure 4:**
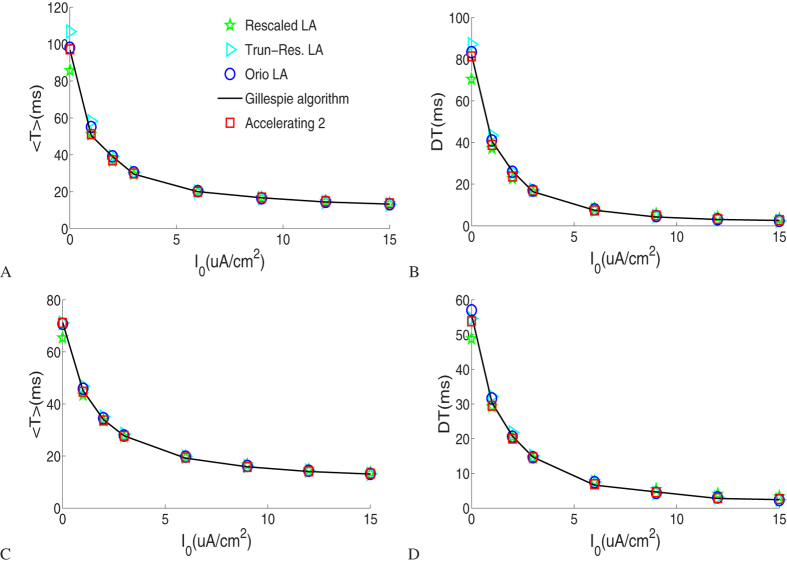
Comparison of the means and standard deviations of the first 10000 interspike intervals as a function of *I*_0_. (**A**,**B**) Results for *I*_1_ = 0 *uA*/*cm*^2^. (**C**,**D**) Results for *I*_1_ = 1 *uA*/*cm*^2^. Here the total number *N* of *K*^+^ is set to 1800.

Figure 4 shows results under two different synaptic noisy current levels with large potassium channel number *N* = 1800, for which the Identical subunit LA breaks down. There are observable discrepancies between these LA approaches at low DC level without input noise as shown in Fig. 4A, in which channel noise still accounts for major effect on the regular firing, just like Fig. 2 suggests. As *I*_1_ increases just by 1 *uA*/*cm*^2^, the mean ISI is much reduced, which is particularly evident at low DC level. The agreement between the accelerating algorithm 2 and the Gillespie calculation is still very good. In general, noisy stimuli help reduce the mean ISI and have a significant effect on spike firing especially for large channel number at low DC level.

### Membrane Voltage statistics

**Figure 5:**
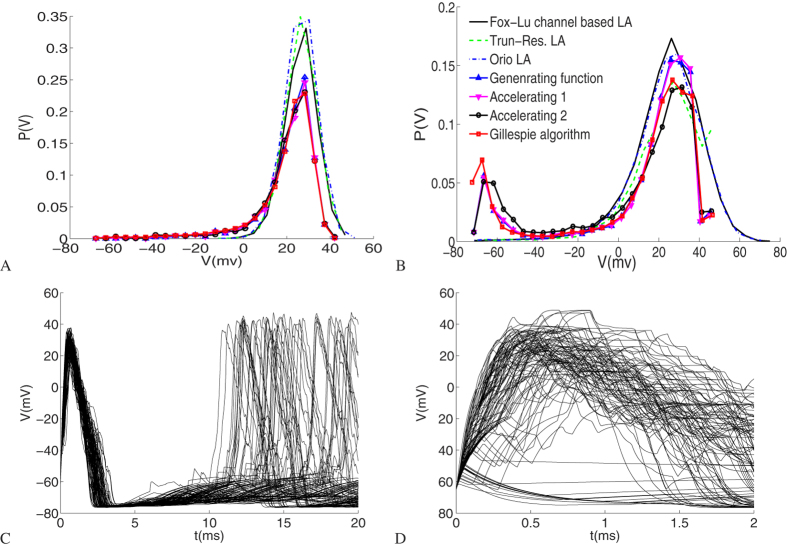
(**A**,**B**) Comparison of the probability distributions of membrane voltage at t = 0.6 ms. (**C**,**D**) The first 100 voltage paths using Gillespie algorithm. (**A**,**C**) The total *K*^+^ channel number *N* is 60, and results are given with the initial condition {*m*_*K*_} = {20, 16, 12, 8, 4}, {*m*_*Na*_} = {40, 32, 28, 24, 20, 16, 12, 8} by the generating function approach, accelerating algorithm 1 and the Gillespie algorithm, while {*m*_*K*_} = {56, 4}, {*m*_*Na*_} = {258, 8} by accelerating algorithm 2, and with the initial fractions of *K*^+^ channel {5, 4, 3, 2, 1}/15, of *Na*^+^ channel {10, 8, 7, 6, 5, 4, 3, 2}/45 by the three channel based Langevin approaches. (**B**,**D**) The total *K*^+^ channel number *N* is 15, and results are given with the initial condition {*m*_*K*_} = {5, 4, 3, 2, 1}, {*m*_*Na*_} = {10, 8, 7, 6, 5, 4, 3, 2} by the generating function approach, accelerating algorithm 1 and the Gillespie algorithm, while {*m*_*K*_} = {14, 1}, {*m*_*Na*_} = {43, 2} by accelerating algorithm 2, and with the initial fractions of *K*^+^ channel {5, 4, 3, 2, 1}/15, of *Na*^+^ channel {10, 8, 7, 6, 5, 4, 3, 2}/45 by the three channel based Langevin approaches.

The membrane voltage statistics are calculated from an ensemble of voltage paths. Figure 5 shows the probability distribution for the membrane voltage at time t = 0.6 ms computed from 10000 runs of the Gillespie algorithm, of the three channel-based Langevin approaches, and of three generating function approaches with the same initial fractions of channel states. Fox-Lu channel based LA is less accurate and slower than Orio LA and Truncated-Restored LA, thus we did not show its computation results in previous discussion.

The results show that our generating function approach captures the distribution function very well when compared with the Gillespie algorithm at two different channel numbers. We can see from Fig. 5A that the membrane voltage distribution ranging from −70–42 *mV* is close to a Gaussian centered around *V* = 27 *mV*, whereas Langevin approaches give nonzero probability only when membrane voltage is larger than −12 *mV*. While in Fig. 5B the distribution has two peaks around −68 *mV* and +27 *mV*, which Langevin approaches fail to reveal. Because the initial numbers of open *K*^+^ and *Na*^+^ channels are not zero, and the initial fractions of the open *K*^+^ and *Na*^+^ channels are the same in Fig. 5A,B, there is supposed to be a spike for any voltage path near *t* = 0.6 *ms* as shown in Fig. 5C, where the membrane voltage is more likely bigger than a minimal spike value. However, what’s interesting is that as the number of *K*^+^ channels decreases to 15, the bistable state appears as shown in Fig. 5B, where another peak locates near *V* = −68 *mV* while the high peak is considerably lower than that in Fig. 5A. From Fig. 5D, we can see that the membrane potential may fail to rise to a value larger than −30 *mV* and quickly fall to the resting level. This observation suggests that the strong channel noise at small channel numbers may sometimes undermine rather than promote spike firing.

### Computation efficiency

**Figure 6:**
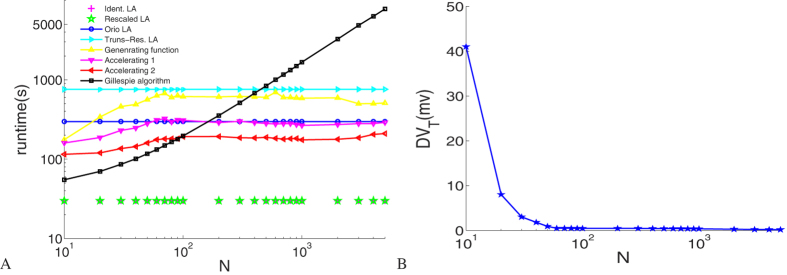
(**A**) Comparison of computing time. The computation is done over a time interval of 40 *s* with a time step of 0.01 *ms*. (**B**) The threshold of the membrane voltage width (*DV*)_*T*_ for different *K*^+^ channel numbers *N*.

Finally, the computational time of these approaches is summarized in Fig. 6A for the case with no input current. Simulations with Matlab were run on a 3.07 GHz quad core Intel Core i7 processor.

Figure 6A shows that generating function approaches are considerably faster than the Gillespie algorithm, particularly for larger channel numbers. The consumed time grows linearly with the number of ion channels in the Gillespie algorithm, depending on the average time between reactions, which can be very small for large channel numbers, while in the generating function approach the computing time remains more or less constant. Channel numbers are irrelevant to the efficiency of the Langevin approach. The two subunit-based Langevin approaches take the same least time compared to others, and the Truncated-Restored LA spends more time mainly for the calculation of root square of the diffusion matrices than the Orio LA. In total, accelerating algorithms 1, 2 are faster than the channel-based LAs, and the generating function approach is faster than the Truncated-Restored LA.

Although the threshold of the membrane voltage width we take empirically monotonically decreases as shown in Fig. 6B, which is consistent with the fact that as channel number increases, channel noise tends to have less and less effect on spike firing. To guarantee the accuracy enough samplings have to be taken, which leads to an extremely slow increase of the computation time in the generating function approach with the total channel number *N*. When *N* is no more than 100, the channel noise seems to be dominating and we choose not to sample membrane voltage.

## Discussion

In this work, we applied the generating function approach to the study of stochastic dynamics of the HH model which is a typical Markovian system, accounting for channel shot noise embedded in the evolution of the discrete ion channel states. We designed a numerical scheme to efficiently solve for the generating function, taking into account the channel noise and voltage fluctuations. At the smooth evolution step, due to the peakedness of the membrane voltage distribution at short times, the random voltage is replaced by its mean and hence the channel state distribution is approximated by a product of multinomial distributions. However, samplings for ion channel states and voltage are needed when a preset width limit of the voltage distribution is reached, which can be estimated with the linear noise approximation. Furthermore, the procedure of how to sample channel numbers from the generating function is explained in detail. In order to make the computation more efficient, we also proposed two accelerating algorithms in which only the number of the open ion channels are sampled since the time evolution of the voltage only depends on this number. The accelerating algorithm 2 further reduces the number of equations by employing a multinomial distribution to approximate the channel states.

Results are compared for different approaches: the generating function, the Gillespie algorithm and the Langevin approaches. We calculated the stationary statistics of the fraction of open channels under voltage clamp. The generating function in this case can be solved exactly for the stationary distribution, and thus the exact expression for the mean and standard deviation of the two open channel states are obtained, whereas most Langevin approaches either overestimate or underestimate the conductance fluctuations except the Truncated-Restored LA. Meanwhile, we studied statistics of action potential spikes at different channel numbers in response to constant and noisy current inputs. Compared to the Gillespie simulation with cumbersome computation, our approach produces result directly in an efficient way. Through our analysis of the statistics of the membrane voltage, it seems that the results from accelerating algorithm 2 match well with those obtained with the Gillespie algorithm, but with much higher efficiency.

It is interesting that with the initial distribution of the ion channel state set up properly, bistable state appears at a particular time when the total channel number is small, which the Langevin approaches fail to reveal. Overall, our simulation results show that the generating function approach provide statistically accurate approximation to the neuronal spiking dynamics for any channel number, while Langevin approaches even break down in some cases.

Finally, we compare the computing time with different numbers of *K*^+^ channels ranging from 10 to 3000, and pointed out that the accelerating algorithms take much less time than the exact Gillespie algorithm, and are also faster than channel-based Langevin approaches. With an empirical selection of the threshold for the membrane voltage width, the new scheme has an accuracy comparable to the Gillespie computation.

Overall, the results from these simulations suggest that the generating function approach is an accurate and fast approximation for discrete-state Markov chain models. In spite of the complexity and nonlinearity of the noisy action potential propagating along an axon, we expect a good performance of the current technique in the study of the neuronal dynamics. An extension of the current technique to the investigation of noisy signal transduction on other types of networks should also be possible.

## Methods

### The discrete HH model

According to the HH model which regards the cell membrane as a capacitor, the membrane potential *V* is governed by


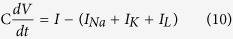


where *C* = 1 *uF*/*cm*^2^ is the membrane capacitance, and *I* is the membrane current. *I*_*Na*_, *I*_*K*_, *I*_*L*_ are the currents of the sodium, potassium, and leakage channels, respectively, and given by


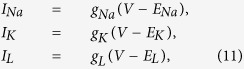


where *E*_*Na*_ = 50 *mV*, *E*_*K*_ = −77 *mV*, *E*_*L*_ = −54.3 *mV* are the reversal potentials of the sodium, potassium, and leakage channels, respectively; *g*_*L*_ = 0.3 *ms*/*cm*^2^, *g*_*Na*_, *g*_*K*_ are their conductances.

As well known, there are four identical and independent *n* subunits for each *K*^+^ channel and three identical and independent *m* subunits and one *h* subunit for each *Na*^+^ channel. Here a single subunit can be in one of the two configurations, open (*O*) or closed (*C*) at time *t*. They convert to each other in a random way more explicitly,


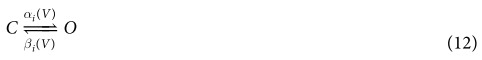


where *α*_*i*_, *β*_*i*_ are voltage-dependent opening and closing rates, and *i* denotes different types of subunits.

Only when these subunits are all open in a channel, is the channel open. Generally, one defines the conductance based on the fraction of open channels. The *K*^+^ conductance is 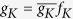, where *f*_*K*_ is the fraction of *K*^+^ channels that are open, and 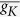 is the maximal conductance of potassium channels. With the knowledge of open channel number 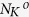 among the total number *N*_*K*_ for *K*^+^ channels, *f*_*K*_ is given by 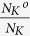, where 
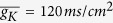
, 
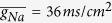
. Similar expressions hold for *Na*^+^ channels. In the discrete description of channels, the voltage equation becomes


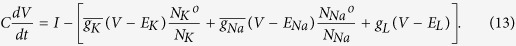


**Figure 7:**
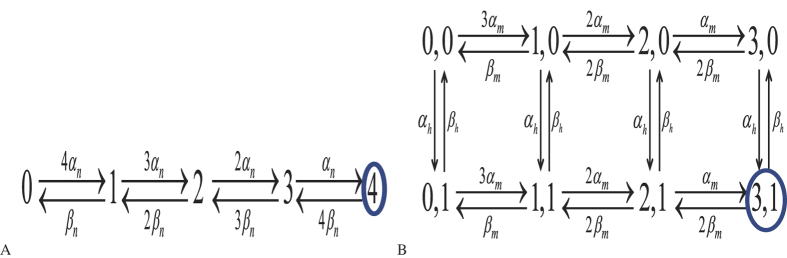
The transition diagram for the *K*^+^ channel (**A**) and for the *Na*^+^ channel (**B**). The numbers in these nodes stand for the numbers of open subunits. (**A**) The number marked with circle is the state in which all four subunits are open. (**B**) A sodium channel is open only when it is in state (3, 1), in which represents the three m subunits and one h subunit are all open.

For the *K*^+^ channel the four *n* subunits define a transition diagram of five channel states, while for *Na*^+^ channel the three *m* subunits and one *h* subunit define a transition diagram of eight channel states. The associated Markov kinetic scheme is depicted in Fig. 7.

Here the voltage-dependent transition rates read^1^


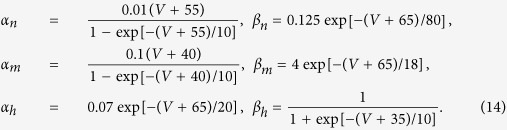


For subunits *m* and *n*, the opening rates increase and the closing rates decrease as the membrane voltage increases, while the opposite is true for subunit *h*.

Individual trajectories of this discrete-state Markov process can be generated exactly using the Gillespie algorithm^28^, in which the waiting time between transitions is sampled from an exponential distribution and the channel number in each state is updated once a specific transition is selected. However the transition rates depend on the membrane voltage, and therefore change over time. If the time between the transition from one state to another is far less than the characteristic voltage variation time, the voltage can be assumed to be fixed during transitions.

A Markov process can be described with master equation, which directly evolves probability distribution in the state space of a system based on specific interstate transition rates. The usually large set of master equations could be recast into a QFT form in which the probability evolution is governed by a wave equation while the field theoretic formulation is equivalent to a generating function approach^40^.

For example, for an elementary reaction such as 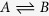, we denote by *P*(*m*, *n*) the probability of having *m* A’s and *n* B’s, the master equation is


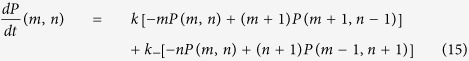


where *k* and *k*_−_ are the forward and backward reaction rates. If the total number of *A*, *B* is large, there would be many equations, each for a particular (*m*, *n*) state. However, they can be transformed into one single PDE for the generating function Ψ(*x*, *y*, *t*) = ∑_*m*,*n*_*P*(*m*, *n*, *t*)*x*^*m*^*y*^*n*^,


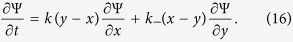


The exact analytic solution can be obtained using the method of characteristics. A solution of generating function eq. (16) reads


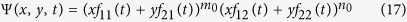


where *m*_0_ and *n*_0_ are initial numbers of *A* and *B*. *x* and *y* are just symbolic variables of *A* and *B*, and *f*_*ij*_ satisfies the equation


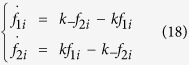


and the initial condition *f*(0) = 1, being an identity matrix.

### Linear noise approximation

All the computation and analysis are based on eq. (4) about the stochastic membrane voltage dynamics with the channel based description, and we could substitute the local 〈*V*〉 for *V* till the width of voltage distribution meets the threshold. Linear noise approximation may be used to compute the width of membrane voltage distribution. Firstly, we assume that


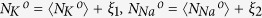


as in the linear noise approximation^36^, with the Gaussian white noise specified by


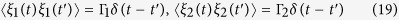


where Γ_1_ and Γ_1_ are defined in eqs (5 and 6), leading to a Langevin equation of *V*


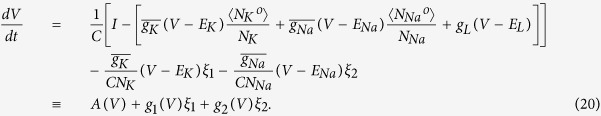


The corresponding Fokker-Planck equation is


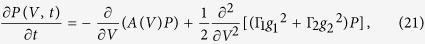


from which one obtains equations for the first and second moment of *V*


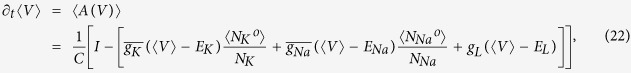



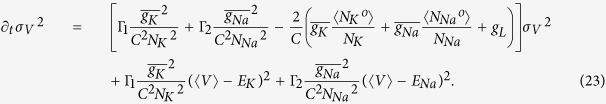


## Additional Information

**How to cite this article**: Ling, A. *et al*. Channel based generating function approach to the stochastic Hodgkin-Huxley neuronal system. *Sci. Rep.* **6**, 22662; doi: 10.1038/srep22662 (2016).
